# Supplementary material for: Computational Identification and 3D Morphological Characterization of Renal Glomeruli in Optically Cleared Murine Kidneys
Source: Sensors (Basel). 2021 Nov 9;21(22):7440. doi: 10.3390/s21227440 (PMC8620055; doi:10.3390/s21227440)
Supplement: Supplementary file 1 [file sensors-21-07440-s001.zip › sensors-1423291-supplementary.pdf]

Article supplementary material

Image processing commands and functions

The following document is a step-by-step description of the commands and functions that have been applied for image processing using ImageJ/Fiji software (ImageJ 2.1.0/1.53h/Java 1.8.0_66 (64-bit)).

Downloading *3D Suites* plugin is mandatory to fully perform the following instructions.

After each step, the image should be saved as a new TIFF file.

1. Image conversion and calibration

The stack is converted to 8-bits format using the following functions: *Image > Type > 8-bit*

The stack is calibrated using the following functions: *Image>Properties…>* The voxel width, height and depth are entered.

2. Grey-level segmentation

The extraction of the 2 structures of interest is performed by a segmentation technique based on 2 greyscale-defined thresholds. The threshold values are determined from the histogram of frequency distribution of the voxel greyscale values.

The histogram is generated using the following functions: *Analyze>Histogram>* Click *'Yes'* to consider all the images of the stack. The histogram hence appears in a new window.

A threshold is applied using the following functions: *Image>Adjust>Threshold…>* It should be checked that only '*Dark background*' and '*Stack histogram*' are ticked. The minimum threshold value is set using the command >*Apply*> selecting '*Default*' in *Method* and '*Dark*' in *Background*, then ticking '*Black background (of binary masks)'" >OK*

3. Filtering

A 3D median filter is applied using the following functions: *Process>Filters>Median 3D…>* Enter *'3'* as a value for x, y and z radii.

4. 3D Manager Options

The isolation of the glomeruli is performed using the *3D Manager* function of the *3D Suites* plugin.

First, the function had to be programmed with the following functions: *Plugins> 3D> 3D Manager Options>* In *'Measurements'* tick the boxes *'Volume (unit)', 'Surface (unit)', 'Compactness', 'Fit Ellipse'>* In '*Options'* tick the boxes '*Use 32-bits image for segmentation (nb objects > 65 535'* and '*Sync with selected 3D viewer'>* Enter, respectively *'10'* and *'1.8'* as values for *'Distance between centers (pixel)* and *'Distance max contact'*

5. 3D volume segmentation

The isolation of the glomeruli is performed in 3 stages using the following functions: *Plugins> 3D> 3D Manager> 'RoiManager3D'*. '*RoiManager*' window is open.

The first stage consisting in the elimination of the small non-glomerular labeled objects based on a 3D volume segmentation is performed using the following steps:

- *a.* A 32-bit analyzable version of the filtered imaged is generated by using the following command: Click '*3D Segmentation'* and set '*Low Threshold'* value at '*1'*.
- *b.* Close the previous image.

c. The objects on the image are added individually in the '*RoiManager3D*' using the following command: Click '*Add Image*'.
d. A table with the volume information of each object is generated using the following command: '*Measure 3D*'.
e. The table generated is saved using the following command that: '*Menu*'> '*Save*'.
f. Once the volume threshold using the '*Vol (pix)*' column is obtained, the objects on the table are classified according to their volume by using the following command: Click on '*Vol (pix)*' column.
g. The objects with a volume equal or higher to the volume threshold are selected on the table, and they are shown on the '*RoiManager3D*' window using the following command on the table window: '*Menu*'> '*Show Objects*'.
h. In order to isolate these objects without using a high-capacity computer, and thus avoiding time waste, first, the image is converted to 8-bits format using the following functions: *Image* > *Type* > *8-bit*; and second, the large objects are erased from the stack using the following command '*RoiManager3D*' window: '*Erase*'.
i. The resulting image is saved and the '*3D Manager*' windows are closed.
j. A threshold is applied on the image to binarize it using the following functions: *Image*>*Adjust*>*Threshold…*> It should be checked that only '*Dark background*' and '*Stack histogram*' are ticked. The minimum threshold value is set at '*1*' using the command >*Apply*> selecting '*Default*' in *Method* and '*Dark*' in *Background*, then ticking '*Black background (of binary masks)*'" >*OK*.
k. The initial filtered image is open.
l. The image eliminating the small non-glomerular labeled objects is obtained by fusing the filtered image to the last obtained image using the following commands: *Process*> *Image Calculator…* > Select the initial filtered image as first and the last obtained image as second image > Choose the '*Subtract*' operation > Tick only '*Create new window*' > OK.
m. The resulting image is the 3D volume segmentation result.

6. 3D shape segmentation

The second stage consisting in the elimination of the large non-glomerular labeled objects based on a 3D shape segmentation is performed using the following steps:

a. The '*3D Manager*' *is* opened by using the following function: *Plugins*> *3D*> *3D Manager*> '*RoiManager3D*' window is open.
b. Steps *4.a to 4.e* are repeated.
c. Once the 'RatioVolEllipsoid' threshold using the '*RatioVolEllipsoid*' column is obtained, the objects on the table are classified on their volume by using the following command: Click on '*RatioVolEllipsoid*' column.
d. The objects with a 'RatioVolEllipsoid' equal or inferior to the 'RatioVolEllipsoid' threshold are selected on the table, and they are shown on the '*RoiManager3D*' window using the following command on the table window: '*Menu*'> '*Show Objects*'.
e. The objects are erased from the stack using the following command '*RoiManager3D*' window: '*Erase*'.
f. The resulting image is saved and the '*3D Manager*' windows are kept open.

7. *Artefacts elimination*

The last stage consisting in the elimination of the artefactual objects is performed using the following steps:

a. On the table, the objects with a volume inferior to 10,000 pixels are selected and they are shown on the *'RoiManager3D'* window using the following command on the table window: *'Menu'> 'Show Objects'*.

b. The objects are erased from the stack using the following command *'RoiManager3D'* window: *'Erase'*.

c. A final table with all the desired information is generated using the following command: *'Measure 3D'*.

d. The generated table is saved using the following command that: *'Menu'> 'Save'*.

e. The final image is saved and the *'3D Manager'* windows are closed.
